# Incidence and Long-Term Survival of Spontaneous Intracerebral Hemorrhage Over Time: A Systematic Review and Meta-Analysis

**DOI:** 10.3389/fneur.2022.819737

**Published:** 2022-03-10

**Authors:** Xianqi Li, Li Zhang, Charles D. A. Wolfe, Yanzhong Wang

**Affiliations:** ^1^School of Life Course and Population Sciences, King's College London, London, United Kingdom; ^2^National Institute for Health Research (NIHR) Biomedical Research Centre (BRC), Guy's and St Thomas' NHS Foundation Trust and King's College London, London, United Kingdom; ^3^NIHR Applied Research Collaboration (ARC) South London, London, United Kingdom

**Keywords:** intracerebral hemorrhage, stroke, incidence, survival, systematic review, meta-analysis

## Abstract

**Background and Purpose:**

Recent epidemiological data indicate that the absolute number of hemorrhagic stroke cases increased by 47% between 1990 and 2010 and continued to cause high rates of death and disability. The last systematic review and meta-analysis of incidence and long-term survival of intracerebral hemorrhage (ICH) were published 11 and 7 years ago, respectively, and lacked comparison between different income groups, therefore, a more up to date analysis is needed. We aim to investigate the ICH incidence and long-term survival data in countries of different income groups.

**Materials Methods:**

We systematically searched Ovid Medline for population-based longitudinal studies of first-ever spontaneous ICH published from January 2000 to December 2020. We performed meta-analyses on the incidence and survival rate in countries of 4 different income groups with random-effects models (severe inconsistency). The *I*^2^ was used to measure the heterogeneity. Heterogeneity was further investigated by conducting the meta-regression on the study mid-year. Time trends of the survival rate were assessed by weighted linear regression.

**Results:**

We identified 84 eligible papers, including 68 publications reporting incidence and 24 publications on the survival rate. The pooled incidence of ICH per 100,000 per person-years was 26.47 (95% *CI*: 21.84–32.07) worldwide, 25.9 (95% *CI*: 22.63–29.63) in high-income countries (HIC), 28.45 (95% *CI*: 15.90–50.88) in upper-middle-income countries, and 31.73 (95% *CI*: 18.41–54.7) in lower-middle-income countries. The 1-year pooled survival rate was from 50% (95% *CI*: 47–54%; *n* = 4,380) worldwide to 50% (95% *CI*: 47–54%) in HIC, and 46% (95% *CI*: 38–55%) in upper-middle income countries. The 5-year pooled survival rate was 41% (95% *CI*: 35–48%; *n* = 864) worldwide, 41% (95% *CI*: 32–50%) in high-income and upper-middle countries. No publications were found reporting the long-term survival in lower-middle-income and low-income countries. No time trends in incidence or survival were found by meta-regression.

**Conclusion:**

The pooled ICH incidence was highest in lower-middle-income countries. About half of ICH patients survived 1 year, and about two-fifths survived 5 years. Reliable population-based studies estimating the ICH incidence and long-term survival in low-income and low-middle-income countries are needed to help prevention of ICH.

**Systematic Review Registration:**

https://www.crd.york.ac.uk/prospero/display_record.php?RecordID=170140, PROSPERO CRD42020170140.

## Introduction

Stroke pathological subtypes include ischemic stroke and hemorrhagic stroke [primary intracerebral hemorrhage (ICH) and subarachnoid hemorrhage (SAH)]. Non-traumatic (spontaneous) ICH is caused by the rupture of small penetrating arteries in the brain and has high mortality and severe disability (1). ICH accounts for 10–15% of strokes worldwide, approximately 20% in low- and middle-income (LMIC) countries and 10% in high-income countries (HIC) (2). According to Global Burden of Disease (GBD) 2010, the absolute number of hemorrhagic stroke cases increased 47% between 1990 and 2010 and caused about 62.8 million disability-adjusted life years (DALYs) lost (86% in LMIC), a significant regional difference in incidence was also found (3).

Long-term prospective population-based studies can provide the most reliable data on stroke incidence. The last systematic review and meta-analysis of population-based studies on ICH incidence were published 11 years ago. Charlotte van Asch et al. found that the worldwide ICH incidence has not decreased over time (4). The overall stroke incidence in low- to middle-income countries has exceeded 20% more than that of high-income countries; however, the comparison of ICH incidence in different income groups remains unclear (2). The last meta-analysis of long-term survival (1 year or more) after ICH was published 7 years ago (5). However, they did not report ICH survival in countries of different income levels. In addition, the pooled estimates of incidence and survival rate in those reviews spanned about 40 years of the study period, which may reduce the comparability and reliability. Since then, several new population-based studies have been published, resulting in a need to update systematic reviews. We, therefore, carried out this systematic review and meta-analysis to update the incidence and long-term survival data of ICH in various income groups.

## Methods

The systematic review and meta-analysis were conducted and reported according to the Preferred Reporting Items for Systematic Reviews and Meta-analyses (PRISMA), Meta-analysis of Observational Studies in Epidemiology (MOOSE), and this study has been registered on PROSPERO (CRD42020170140) (6, 7). The study protocol was not previously published.

### Search Strategy

We searched Ovid Medline and PubMed database for population-based longitudinal studies of spontaneous ICH from January 2000 to December 2020, using the comprehensive electronic literature search strategy with a combination of different keywords “population,” “population-based,” “regional,” “community,” “stroke register,” “incidence,” “fatality,” “mortality,” “trend,” “survival,” “h(a)emorrhagic stroke,” “intracranial h(a)emorrhage,” “cerebral h(a)emorrhage,” “intracerebral h(a)emorrhage,” “haemorrhage,” or “h(a)ematoma.” The detailed searching strategy is in Appendix 1 in Supplementary Material. Further eligible studies were reviewed through reference lists and advice from experts to avoid missing any literature.

### Eligibility Criteria

We included population-based studies reporting incidence or long-term survival (1 year and 5 years) of lifetime ICH. We only included prospective studies in the incidence analysis because there are numbers of new cases of ICH in the target population over the specified study period, which enable us to calculate incidence. However, we included both prospective and retrospective studies in the survival analysis. Only papers published in English were included.

We excluded studies if: (1) they only investigated the >85 years old and pediatric/adolescent (<18 years old) population; (2) hospital-based studies; (3) the data cannot be extracted for analysis; (4) ICH was not an incident event; (5) they included non-spontaneous or traumatic ICH cases into the ICH group; (6) they included subarachnoid hemorrhage or pure intraventricular hemorrhage into the ICH group; (7) there was no clear illustration of most ICH case ascertainment using imaging or pathological methods (autopsy) since the imaging techniques for identifying stroke subtypes were widely used since the 1990s; (8) over half of the study period was before 1995; (9) the total study sample size less than 20, which means the cohort or population size in incidence studies and ICH patient numbers in survival studies; and (10) it was not first-ever ICH.

### Study Selection

Two investigators (XL and LZ) independently undertook the search and screening. One investigator (XL) first screened all titles and abstracts after removing duplicate papers. The remaining studies were read in full for eligibility assessment to be included in the final analysis by the second investigator (LZ). The disagreements and uncertainties in the process were resolved by discussion with a third reviewer (YW).

### Data Extraction

We extracted each study period accurate to month if possible and calculated the study midyear, which is used to represent the study time for each independent study. If the study period was an even number, we assessed the midyear as the next year of the middle time point (e.g., study period: 2001, 2002, 2003, 2004, midyear: 2003). Additionally, we assessed the number of incident ICH cases in the study period, number of person-years, number of survivors after 1 year or 5 years, and the number of study population if applicable. We calculated person-years by multiplying the number of the study population and total study years together if it was not directly reported in the study. If several published papers were from the same longitudinal study (e.g., stroke registry), we extracted data from papers with the longest person-years or papers reporting different periods of the same stroke study. We recorded the age information for studies reporting incidence. Countries and their income levels {(I) high income, (II) upper-middle income, (III) lower to middle income, (IV) low income according to the World Bank's country classification (8)} were recorded.

### Quality Assessment of Studies

The Newcastle Ottawa scale (NOS) (9) was used to evaluate the quality of the cohort study. The evaluation criteria initially include (I) selection: (1) the representativeness of the exposed cohort, (2) selection of the non-exposed cohort, (3) ascertainment of exposure, (4) outcome of interest was not present at the start of the study; (II) (5&6) comparability of exposed and non-exposed cohorts; (III) outcome: (7) assessment of outcome, (8) length, and (9) adequacy of follow up. However, due to the nature of our eligible studies, there is no information about the exposed cohort. Therefore we use (1), (4), (7), (8), and (9) to perform the quality assessment; the maximum score is 5, which means very high quality, 4 means high quality, 3 means acceptable quality, and 1 and 2 means relatively low quality.

### Statistical Methods

The incidence was computed as the number of ICH cases per 100,000 person-years. Survival rate was calculated as the proportion of survivors of ICH at 1 year or 5 years. Incidences and survival rates were separately visualized in world maps. Different shades of color means the density and the gray area indicates no data. Median was used if more than one study reported incidences or survival rates in one country. We performed meta-analyses for both incidences, 1-year survival rate and 5-year survival rate using statistical software R.3.6.3. The *I*^2^ and Cochran's *Q*-test were used to measure the heterogeneity across studies. About 25, 50, and 75% were considered the cut-off of *I*^2^values that indicate the low, moderate, and high degree of heterogeneity, respectively. A random-effects model with DerSimonian and Laird method was used in the meta-analyses of incidence and survival rate of ICH to generate a pooled estimate because of the high heterogeneity across studies. Subgroup analyses of countries on different income levels and meta-regression of study midyear were conducted to identify the source of heterogeneity. A weighted linear regression was used to assess the temporal trend of the survival between survival and the mid-year of study periods; the reverse of standard error in each study was used as weight. Sensitivity analyses were performed to examine the impact of any single study on the pooled estimate result. Funnel plots were used to evaluate the publication bias. A *p* < 0.05 was considered statistically significant.

## Results

**Figure 1:**
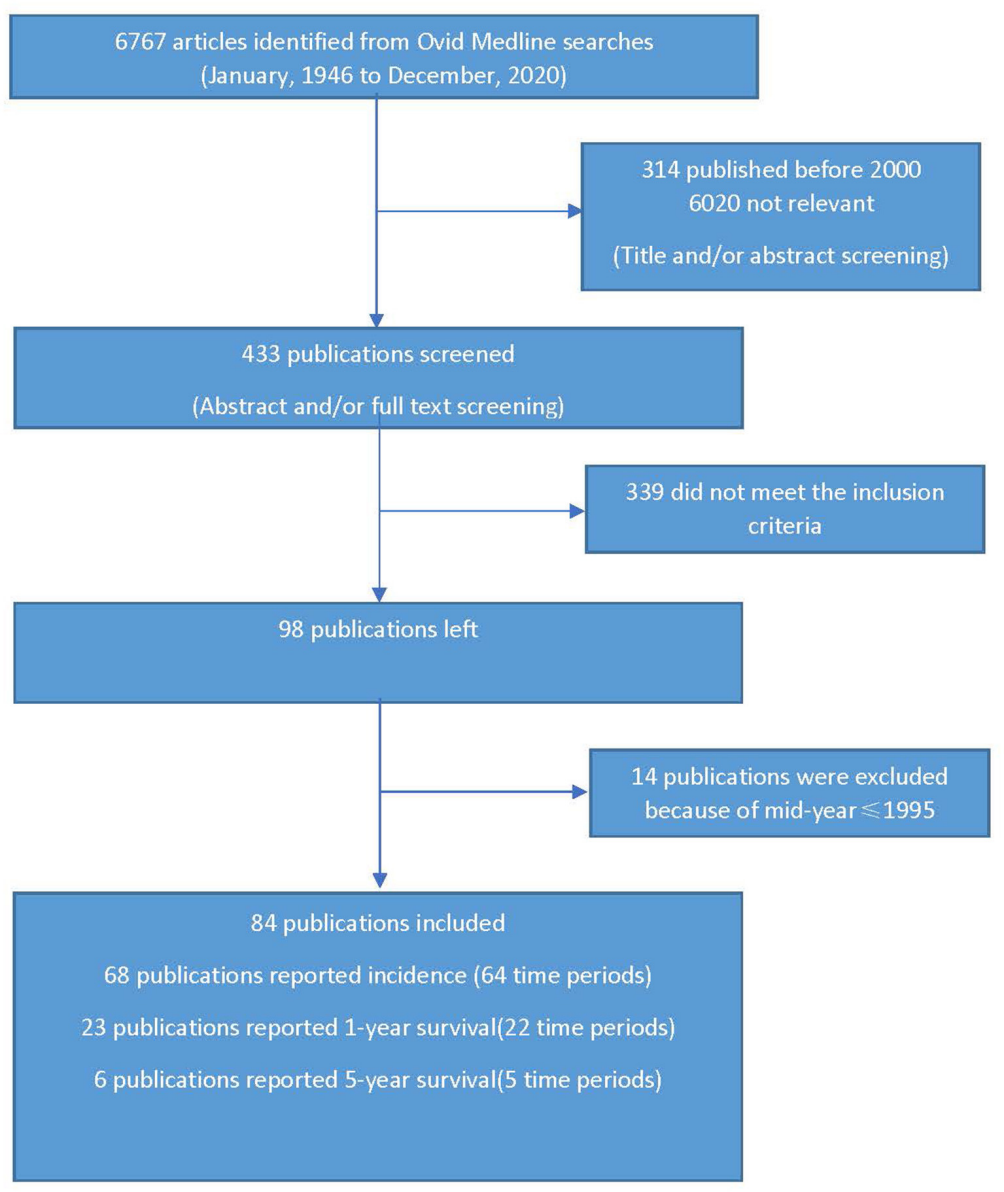
Literature search.

We initially retrieved 6,767 potentially relevant publications (Figure 1). The titles, abstracts, and publication time were scanned for appropriateness for our review, and those not relevant were removed (6,334). The abstracts and full text of the remaining 433 studies were reviewed to meet the inclusion criteria and 339 publications were removed. Data of the remaining 98 manuscripts were then extracted, which led to the exclusion of a further 14 manuscripts whose mid-year of study period did not meet the eligibility criteria. Thus, 84 eligible population-based studies in 25 countries were available for analysis, of which 68 articles reported 64 time periods about incidence, 23 articles reported 22 time periods about 1-year survival, 6 articles reported 5 time periods about 5-year survival (Tables 1, 2; Figure 5). There are 8 publications reporting both incidence and survival. All these eligible papers were published after 2000, in which the study time periods ranged from 1987 to 2017. One publication may report several time periods of incidence or survival rate (studies with long follow-up time). Several publications may report the same time periods (publications from the same database or stroke center/registry). Therefore we use “time periods” other than “studies” to specifically represent the parameter included in this meta-analysis.

**Table 1:** Characteristics of studies reporting incidences on intracerebral hemorrhage (ICH).

**References**	**Country**	**Income level**	**Study period**	**Mid-year of study**	**ICH patients (n)/person years**	**Incidence rate (per 100,000 per year)**	**Age range**
Wolfe et al. (10)	Dijon, France	High	1995.01–1997.12	1996	37/429,264	8.62	No
Thrift et al. (11)	Melbourne, Australia	High	1996.05–1997.04	1996	40/429,264	29.89	No
Di Carlo et al. (12)	Vibo Valentina, Italy	High	1996.01–1996.12	1996	62/179,186	34.60	No
Sacco et al. (13)	l'Aquila, Italy	High	1994.01–1998.12	1996	549/1,488,225	36.89	No
Marini et al. (14)							
D'Alessandro et al. (15)	Valle d'Aosta, Italy	High	1996.11–1997.10	1997	36/118,723	30.32	No
Kita et al. (16)	Takashima, Japan	High	1996–1998	1997	73/166,353	43.88	No
Ishikawa et al. (17)	12 districts, Japan	High	1992.04–2005.12	1998	102/131,718	77.44	No
Zhang et al. (18)	China	Upper-middle	1996–2000	1998	2,275/5,657,595	40.21	>25
Smadja et al. (19)	Martinique	High	1998.06–1999.05	1998	83/360000	23.06	No
Thrift et al. (20)	Melbourne, Australia	High	1997.05–1999.04	1998	151/613,262	24.62	No
Correia et al. (21)	North Portugal	High	1998.10–2000.09	1999	108/243,116	44.42	No
Appelros et al. (22)	Örebro, Sweden	High	1999.02–2000.01	1999	44/123503	35.63	No
Syme et al. (23)	Scotland, UK	High	1998.10–2000.09	1999	50/212,704	23.51	No
Suzuki et al. (24)	Akita, Japan	High	1995–2004	1999	7,423/12,000,000	61.86	No
Kolominsky-Rabas et al. (25)	Erlangen, Germany	High	1995.01–2002.12	1999	194/841,312	23.06	No
Correia et al. (26)	Porto, Portugal	High	1998–2000	1999	78/172,046	45.34	No
Islam et al. (27)	Perth, Australia	High	2000.02–2001.02	2000	19/143,417	13.25	No
Heuschmann et al. (28)	South London, UK	High	1995–2004	2000	395/2,701,909	14.62	No
Smeeton et al. (29)							
Giroud et al. (30)	Dijon, France	High	1987.01–2012.12	2000	530/3,896,484	13.60	No
Kita et al. (16)	Takashima, Japan	High	1999–2001	2000	89/166,353	53.50	No
Vaartjes et al. (31)	Netherlands	High	2000	2000	3,791/13,657,649	27.76	No
Hallstrom et al. (32)	Lund, Sweden	High	2001.03–2002.02	2001	46/235,505	19.53	>15
Hallstrom et al. (33)							
Lavados et al. (34)	Iquique, Chile	High	2000.07–2002.06	2001	69/396,712	17.39	No
Lavados et al. (35)							
Feigin et al. (36)	Auckland, New Zealand	High	2002.03–2003.02	2002	177/897,882	19.71	>15
Corbin et al. (37)	Barbados	High	2001.10–2002.09	2002	42/239,068	17.57	No
Manobianca et al. (38)	Puglia, Italy	High	2001.01–2002.12	2002	24/77,474	30.98	No
Vibo et al. (39)	Tartu, Estonia	High	2001.12–2003.11	2002	57/202,244	28.18	No
Li et al. (40)	Tianjin, China	Upper-middle	1999–2005	2002	53/100,297	52.84	No
Wang et al. (41)							
Wang et al. (42)							
Manobianca et al. (43)	Puglia, Italy	Upper-middle	2001.01–2002.12	2002	24/77,470	30.98	No
Dalal et al. (44)	Oxford, UK	High	2002.04–2004.03	2003	34/273,318	12.44	No
Rothwell et al. (45)							
Minelli et al. (46)	Matão, Brazil	Upper-middle	2003.11–2004.10	2004	11/75,053	14.66	No
Carlsson et al. (47)	Tromsø, Norway	High	1995.01–2012.12	2004	226/453,152	49.87	≥30
Kleindorfer et al. (48)	5 counties, USA	High	2005.01–2005.12	2005	321/1,319,856	24.32	No
Kissela et al. (49)							
Corso et al. (50)	Aosta, Italy	High	2004.01–2005.12	2005	58/247,496	23.43	No
Groppo et al. (51)	Ferrara, Italy	High	2002–2007	2005	10/323,250	3.09	15-44
Dalal et al. (44)	Mumbai, India	Lower-middle	2005.01–2006.12	2006	67/313,722	21.36	>25
Cabral et al. (52)	Joinville, Brazil	Upper-middle	2005–2006	2006	88/791,675	11.12	No
Cabral et al. (53, 54)							
Kelly et al. (55)	Dublin, Ireland	High	2005.12–2006.11	2006	56/294,529	19.01	No
Amiri et al. (56)	Mashhad, Iran	Upper-middle	2006.11–2007.11	2007	90/450,229	19.99	No
Azarpazhooh et al. (57)							
Boden-Albala et al. (58)	Alaska, USA	High	2005.10–2009.10	2007	47/560,000	8.39	No
Kolominsky-Rabas et al. (25)	Erlangen, Germany	High	2003.01–2010.12	2007	200/841,312	23.77	No
Palm et al. (59)	Ludwigshafen, Germany	High	2006.01–2010.12	2008	152/838,285	18.13	No
Janes et al. (60)	Udine, Italy	High	2007.04–2009.03	2008	95/306,624	30.98	No
Pikija et al. (61)	VaraŽdin, Croatia	High	2007.07–2009.06	2008	123/368,230	33.40	No
Li et al. (40)	Tianjin, China	Upper-middle	2006–2012	2009	106/100,157	105.83	No
Wang et al. (41)							
Wang et al. (42)							
Gattellari et al. (62)	New South Wales, Australia	High	2005.01–2013.12	2009	12986/51,185,251	25.37	No
Neelamegam et al. (63)	Penang Island, Malaysia	Upper-middle	2010.04–2011.03	2010	32/197,131	16.23	No
Correia et al. (26)	Porto, Portugal	High	2009–2011	2010	43/204,424	21.03	No
Cabral et al. (52)	Joinville, Brazil	Upper-middle	2010–2011	2011	82/760,172	10.79	No
Stranjalis et al. (64)	The Isle of Lesvos, Greece	High	2010.06–2011.05	2011	52/86,436	60.16	No
Tsivgoulis et al. (65)	Evros, Greece	High	2010–2012	2011	83/119,805	69.28	>20
Takashima et al. (66)	Shiga, Japan	High	2011	2011	551/1,400,745	39.34	No
Samarasekera et al. (67)	Scotland, UK	High	2010.06–2011.05	2011	128/695,335	18.41	≥16
Okon et al. (68)	Akure, Nigeria	Lower-middle	2010.11–2011.10	2011	265/491,033	54.00	No
Sacco et al. (69)	l'Aquila, Italy	High	2011.01–2012.12	2012	115/596,686	19.27	No
Pandian et al. (70)	Ludhiana, India	Lower-middle	2012.03–2013.03	2012	290/1,065,127	27.23	≥18
Olindo et al. (71)	Martinique	High	2011.11–2012.10	2012	84/370,854	22.65	No
Chen et al. (72)	10 areas, China	Upper-middle	2008.01–2017.01	2012	7,440/4,406,274	168.85	35–74
Wang et al. (73)	China	Upper-middle	2013	2013	391/479,044	81.62	≥20
Saliba et al. (74)	Israel	High	2010.01–2017.12	2014	4170/10,730,915	38.86	≥40
Cabral et al. (52)	Joinville, Brazil	Upper-middle	2014–2015	2015	79/789,418	10.01	No
Nzwalo et al. (75)	Algarve, Portugal	High	2015.01–2015.12	2015	82/280,081	29.28	No
Minelli et al. (76)	Matao, Brazil	Upper-middle	2015.08–2016.07	2016	10/78,890	12.68	No
Appelros et al. (77)	Örebro, Sweden	High	2017.01–2017.12	2017	36/150,291	23.95	No

**Table 2:** Characteristics of studies reporting survival rate after 1 year and/or 5 years on ICH.

**Studies**	**Country**	**Income level**	**Study period**	**Mid-year of study**	**ICH patients (n)**	**1-year survivors (n)**	**5-year survivors (n)**
Thrift et al. (11)	Melbourne, Australia	High	1996.05–1997.04	1996	40	20	
Di Carlo et al. (12)	Vibo Valentina, Italy	High	1996.01–1996.12	1996	62	27	
Sacco et al. (13)	L'Aquila, Italy	High	1994–1998	1996	464	225	
Hillen et al. (78)	London, UK	High	1995–2000	1998	222	110	72
Thrift et al. (20)	Melbourne, Australia	High	1997–1999	1998	151	76	
Syme et al. (23)	Southeast Scotland, UK	High	1998.10–2000.09	1999	50	22	
Vibo et al. (39)	Tarsu, Estonia	High	2001–2003	2002	57	26	
Hansen et al. (79)	Southern Sweden	High	1996–2009	2003	323	172	127
Waziry et al. (80)	Rotterdam, Netherlands	High	1991–2015	2003	162	55	
McCormick et al. (81)	London, UK	High	1995–2011	2003	562	297	
van Beijnum et al. (82)	Oxford, UK	High	2002–2007	2005	56	22	
Shoeibi et al. (83)	Mashhad, Iran	Upper-middle	2006.11–2007.11	2007	86	38	
Palm et al. (59)	Ludwigshafen, Germany	High	2006–2010	2008	152	85	
Desikan et al. (84)	London, UK	High	2005–2012	2009	204		106
Farzadfard et al. (85)	Mashhad, Iran	Upper-middle	2006–2012	2009	80	38	31
Samarasekera et al. (67)	Scotland, UK	High	2010.06–2011.05	2010	128	56	
Oie et al. (86)	Norway	High	2008.01–2014.12	2011	452	249	
Tsivgoulis et al. (87)	Southern Greece	High	2010– 2012	2011	83	44	
Nzwalo et al. (88)	Algarve, Portugal	High	2009–2015	2012	545	296	
Olindo et al. (89)	Martinique	High	2011.11–2012.10	2012	84	43	
Takashima et al. (90)	Shiga, Japan	High	2011–2013	2012	551	390	
Sacco et al. (69)	L'Aquila, Italy	High	2011–2012	2012	115	55	
Cabral et al. (91) Cabral et al. (92)	Joinvile, Brazil	Upper-middle	2010.01–2015.12	2013	35	18	16

### Incidence of ICH

Overall, 64 time periods with 45,224 patients of 127,308,087 person-years were included, of which 19 time periods had a number of person-years less than 200,000, 33 time periods from 200,000 to 1,000,000, 8 time periods from 1,000,000 to 10,000,000, and only 4 study periods more than 10,000,000 (Table 1). However, the range of crude incidence varied from 3.09 to 105.83 per 100,000 person-years across studies. The greatest incidence, 168.85 per 100,000 person-years from China, was about 6 times greater than the median incidence (average of the United States and Australia) 24.47 per 100,000 persons-years. Japan has the greatest incidence 77.44 per 100,000 person-years among high-income countries (HIC) and 53.97 per 100,000 person-years in Akure, Nigeria is the greatest in lower-middle income countries. Studies of 12 time periods had an age limitation, of which one study in Ferrara, Italy includes only patients from 15 to 44 years old (51). The sensitivity analysis of the incidence in 52 time periods in studies without age limitation was 25.38 (95% *CI*: 21.97–29.32, *I*^2^ = 99.10%) per 100,000 person-years.

**Figure 2:**
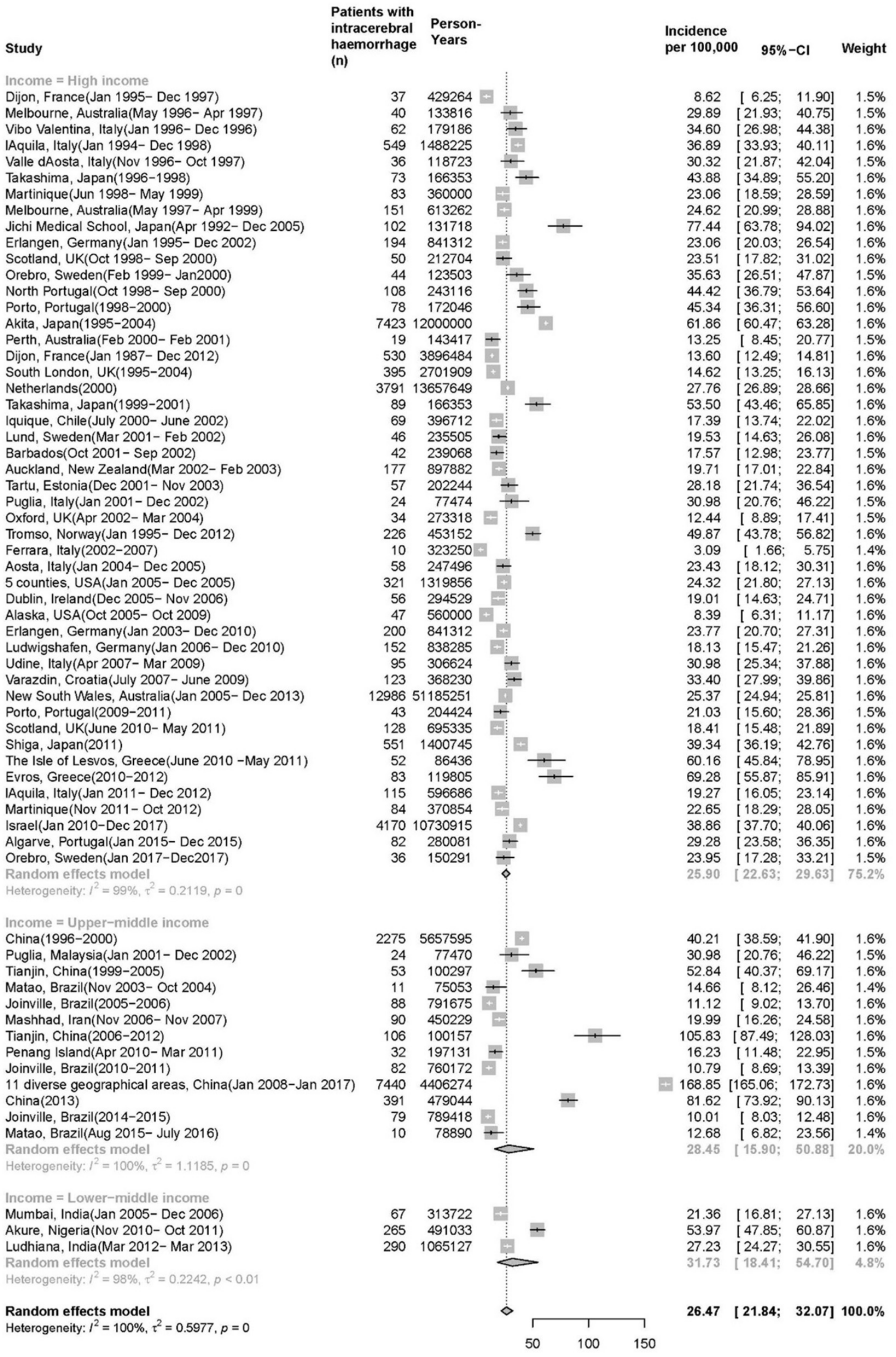
Incidence of intracerebral hemorrhage (ICH).

There are 48 time periods with 33,921 patients and 111,474,800 person-years, 13 time periods with 10,681 patients and 13,963,405 person-years, 3 time periods with 411 patients and 1,869,882 person-years of high-income, upper-middle income and low-middle income countries, respectively (Figure 2). The pooled ICH incidence for 64 time periods was 26.47 per 100,000 persons per year (95% *CI*: 21.84, 32.07), with significant heterogeneity (*I*^2^ = 100%, *p* = 0). The pooled incidence in HIC was 25.90 per 100,000 person-years (95% *CI*: 22.63, 29.63, *I*^2^ = 99%, *p* = 0), which is marginally lower than that of the upper-middle income countries 28.45 per 100,000 person-years (95% *CI*: 15.90, 50.88, *I*^2^ = 100%, p = 0), and the lower-middle income countries has the highest incidence 31.73 per 100,000 person-years (95% *CI*: 18.41, 54.70, *I*^2^= 99%, *p* < 0.01). There are 11 time periods in the upper-middle income group but only from 4 countries (China, Malaysia, Brazil, and Iran).

In meta-regression analysis, after adjusting for incidence data and midyear of each study period, it indicated that midyears are not significantly associated with effect size differences in all studies, and incidence of ICH has not decreased from 1996 to 2017 (annual decrease of 0.0056 per 100,000 person-years, 95% *CI*: −0.0318 to 0.0206, *p* = 0.67, tau^2^ = 0.30). There was either no temporal trend in incidence of HIC from 1995 to 2015 (annual decrease of 0.0060 per 100,000 person-years, 95% *CI*: −0.0383 to 0.0264, *p* = 0.7179, tau^2^ = 0.32).

### Survival After ICH

**Figure 3:**
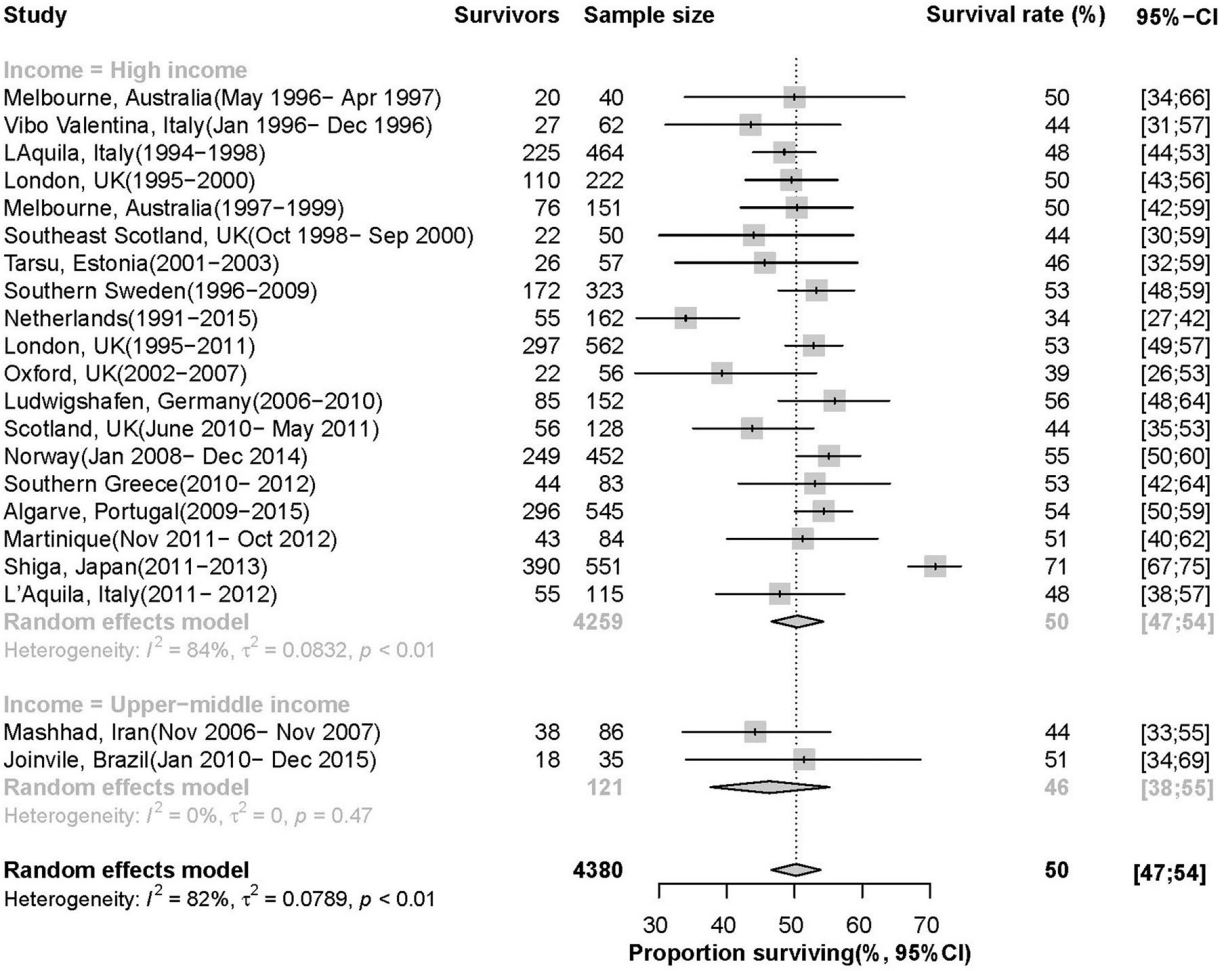
Survival rate of patients 1 year after ICH.

Figure 3 showed that 22 time periods reporting the 1-year survival rate of ICH patients, with 2,326 survivors and 4,380 patients in total, the pooled estimate is 50% (95 *CI*: 47%, 54%, *I*^2^= 82%, *p* < 0.01). The survival rate in HIC is 50% (95 CI: 47%, 54%, *I*^2^= 84%, *p* < 0.01), which is higher than 46% (95 *CI*: 38%, 55%, *I*^2^= 0%, *p* = 0.47) in upper-middle income countries. There was no temporal trend in 1-year survival from 1996 to 2015 (annual increase of 0.6% per year, 95% CI: 0.0–0.12%, *p* = 0.40).

**Figure 4:**
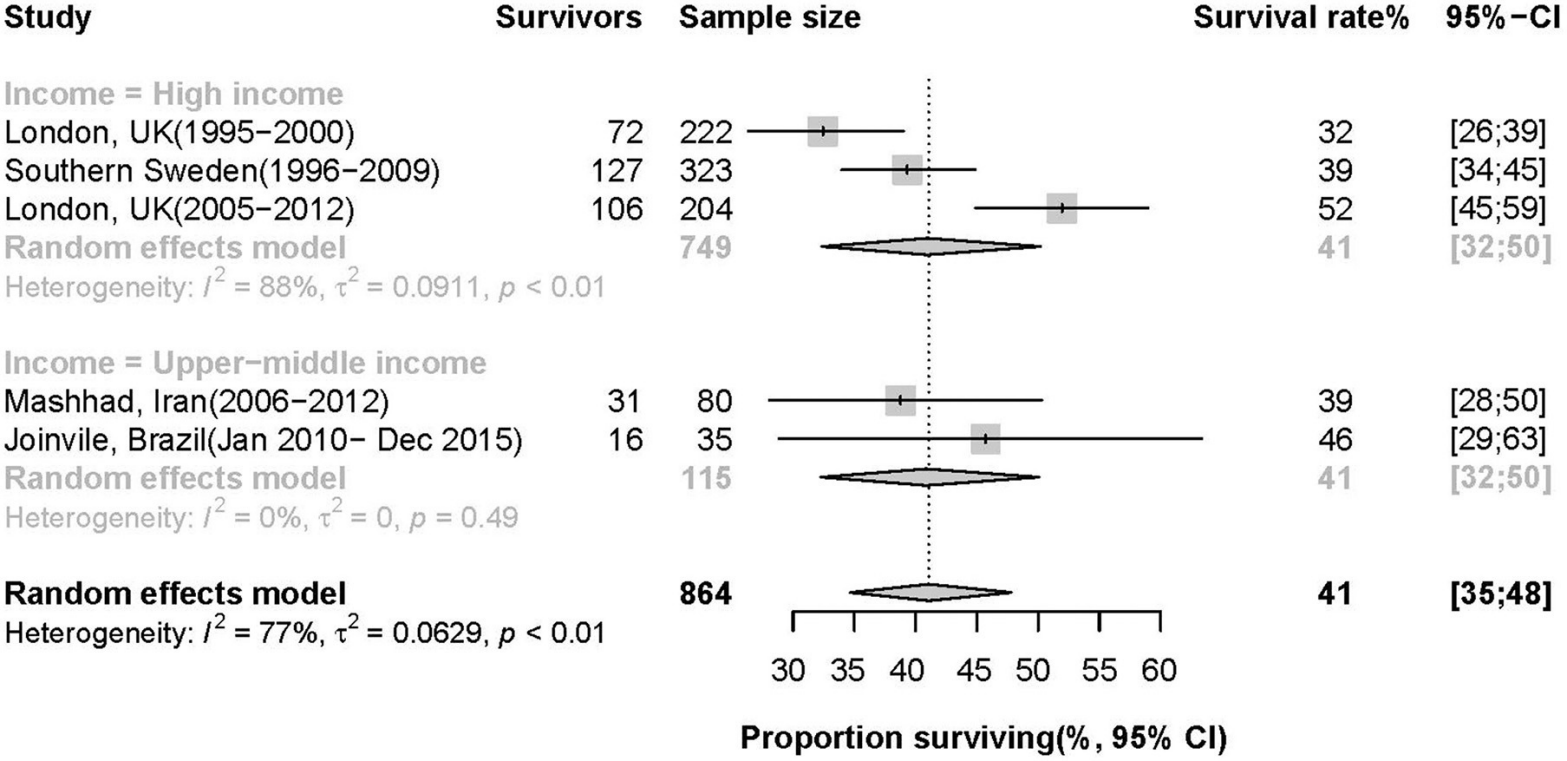
Survival rate of patients 5 years after ICH.

There were 352 survivors of 864 patients with ICH in studies reporting a 5-year survival rate, of which the pooled estimate was 41% (95% *CI*: 35%, 48%, *I*^2^= 99%, *p* = 0.19). The 5-year survival rate varies from 32 to 46% across 5 studies (Figure 4). The two different income groups had the same pooled 5-year survival rate but different degrees of heterogeneity as 41% (95 *CI*: 32%, 50%, *I*^2^= 88%, *p* < 0.01) and 41% (95 *CI*: 32%, 50%, *I*^2^= 0%, *p* = 0.49) in high-income and upper-middle countries, respectively. There was no temporal trend in 5-year survival from 1995 to 2015 (an annual increase of 1.1% per year, 95% *CI*: −0.4 to 2.7%, *p* = 0.11).

**Figure 5:**
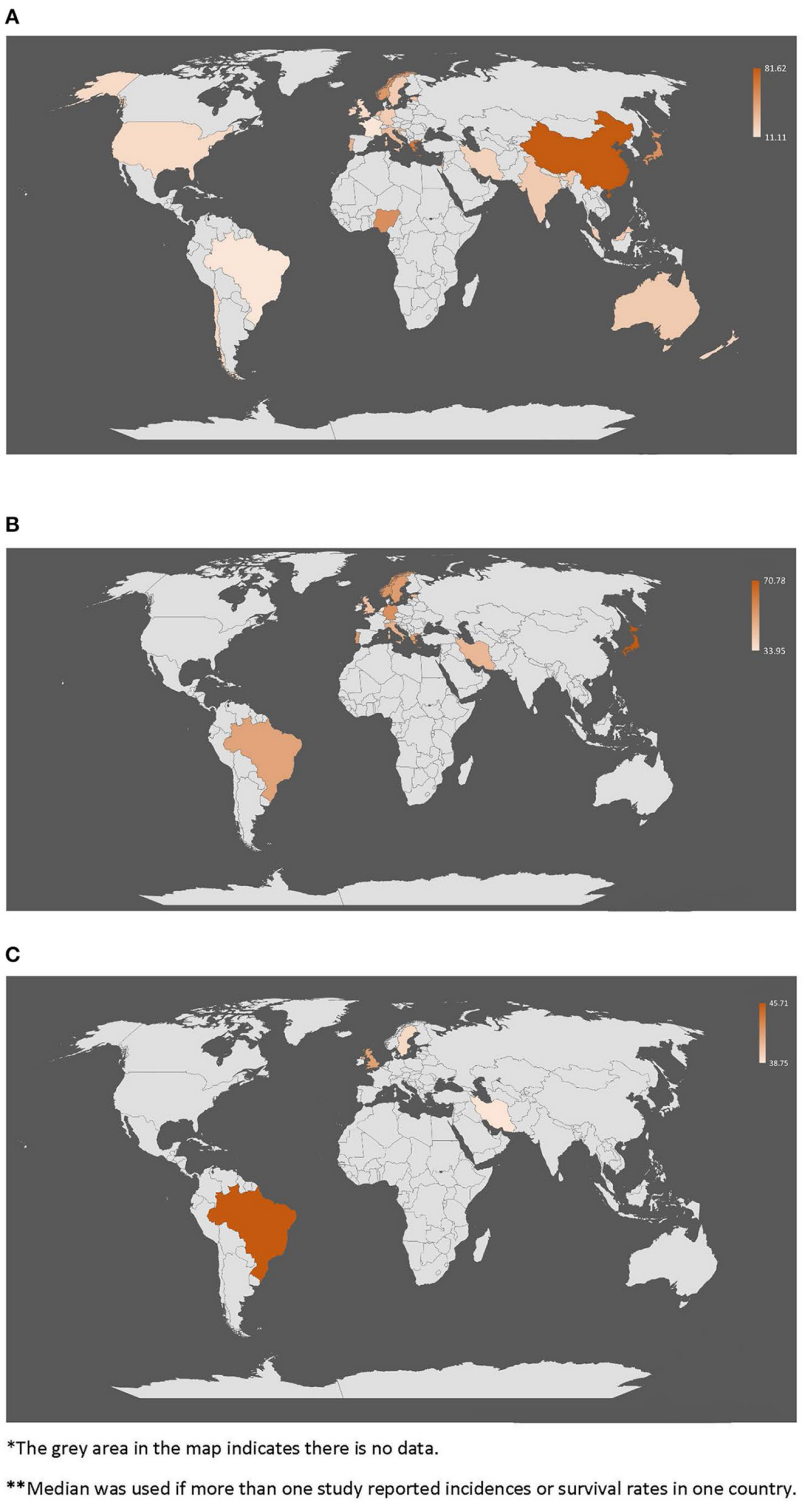
World map * of incidence and 1-year/5-year survival rates **. **(A)** Incidences of intracerebral hemorrhage across the world (per 100,000 per year). **(B)** 1-year survival rate of intracerebral hemorrhage across the world (%). **(C)** 5-year survival rate of intracerebral hemorrhage across the world (%).

### Quality Assessment, Sensitivity Analysis, and Publication Bias

Approximately 90% of the eligible studies were of very high quality (details in Supplementary Material). There were no significant changes in incidence and 1-year survival meta-analyses when any study was removed. Funnel plots of three meta-analyses are slightly asymmetric, indicating modest publication bias of studies (Appendix 3–5 in Supplementary Material).

## Discussion

Overall, this systematic review and meta-analysis of 80 population-based longitudinal cohort publications about the incidence and long-term survival after spontaneous primary ICH found that pooled overall incidence was 26.47 per 100,000 person-years, the 1-year survival rate was 49%, and 5-year survival rate was 41%. The incidence and survival rate have not changed over time.

The pooled overall incidence worldwide in our review was slightly higher than the systematic review and meta-analysis published 11 years ago (26.47 vs. 24.6 per 100,000 person-years) (4). The difference may result from the completion of more population-based stroke studies in recent years in upper-middle countries with high incidences, such as China (18, 40, 42, 72, 73). Neither study found a decrease in ICH incidence worldwide over time (1980–2006 and 1995–2015). However, the Oxford Vascular Study (OXVASC; 2002–2017) found that ICH incidence declined by comparing stroke incidence with Oxfordshire Community Stroke Project (OCSP; 1981–1986) and other population-based studies in HIC (93). They only include studies that can compare incidence with themselves between the 1990s and 2010s, which can avoid other confounding factors. In the United Kingdom, the OXVASC group stated that the fall in ICH incidence under 75 years old between 1981 and 2006 might result from the prevention and management of hypertension (94). However, the South London stroke Register did not find a steady decrease every 2 years from 1995 to 2004 (95).

Intracerebral hemorrhage location may impact the incidence, some studies in high-income groups reported different incidence trend in lobar or deep ICH (69, 94, 96). The OXVASC (94) study and Dijon stroke registry (96) both found the increasing incidence of non-hypertensive lobar ICH in elderly was related to amyloid angiopathy, maybe partly due to the antithrombotic drugs use. Those trends could be different in low-income countries, in which hypertensive ICH is a rising problem because of less hypertension prevention (3).

China and Japan had the highest incidence in upper-middle income and high-income groups, respectively, (17, 40–42, 72), which may indicate that the ICH incidence was associated with ethnicity in addition to income levels. A systematic review stated that the Chinese people were more likely to have ICH than white people (pooled proportion 33 vs. 12%) (97), but a study in New Zealand found no difference between Asian migrants and white people regarding ICH incidence (36). The most effective solution to reduce the stroke incidence is primary prevention (98); the HIC that have the lowest ICH incidence may be a result from the improvement in primary vascular prevention (2). A recent paper about stroke burden in Latin American countries stated that their progress on the prevention was slower than HIC (99).

There are no available population-based studies on the long-term survival after ICH in low-income and lower-middle income countries. However, a systematic review and meta-analysis of hospital-based studies in sub-Saharan Africa (countries with poverty) stated that the overall 30-day survival of overall stroke was much lower compared with western HIC due to weak healthcare systems (100). We found improved long-term survival compared with the systematic review published 7 years ago (1-year survival: 49 vs. 46%, 5-year survival: 41 vs. 29%), they also reported no temporal trend in survival (5). The better long-term survival maybe due to the better management of the independent risk factor (diabetes mellitus and atrial fibrillation) and the more appropriate use of anticoagulation therapy at ICH onset (78, 79).

Brazil, an upper- to middle-income country, has relatively lower incidences (10.01, 10.79, 11.12, 12.68, 14.66 per 100 000 person-years) (46, 52–54, 76) and higher survival rates (1-year survival rate: 51% and 5-year survival rate: 46%) (91, 92) than some European countries in high-income group. The age distribution could be one reason explaining the better results in Brazil. The percentage of the Brazilian population older than 65 is only 6.0%, which ranging from 13.1 to 21% in other studies (20, 23, 30, 45). Older people have a higher risk of being affected by ICH. The mean age in the Joinville study in Brazil was 63 years old, which is lower than 72 and 73 in Oxfordshire and Perth (54, 101–103). Therefore, the younger age in Brazil for first-ever ICH might contribute to a better survival rate than other populations. Other reasons, such as new drugs for secondary prevention and decreased prevalence of smoking (104) in Brazil cannot be accurately measured, especially when compared with the high-income group. Further research can investigate more on better results in Brazil.

Temporal trends on the long-term survival were not found in this study. This is the reason why we analyze the incidence and survival rate of different time periods together. However, several studies report an improvement in case-fatality after ICH across years in the same region. Sipilä et al. found the case fatality of hospital-treated ICH decreased in 2004–2018 in Finland (105). Béjot et al. found that 30-day case fatality of ICH decreased from 40.9 to 29.6% between 1985 to 2011 in Dijon, France (106). A study in Ontario, Canada, reported substantial reductions in ICH case fatality from 2003 to 2017 (107). We included studies from many different regions, with markedly different healthcare systems, across different study periods in the linear regression, this may represent a relevant bias.

We did not include studies that only investigated less than 18 years old and pediatric (<18 years old) population because the population >85 years of age was a strong predictor of death after stroke (108) and stroke is rare in pediatric patients (109), which may seriously bias the pooled estimates. Studies with midyear before 1995 were excluded because of the significant decrease of stroke incidence in the HIC in the last 20 years (2, 93).

### Strengths and Limitations

The main strengths of this study are three-fold. First, this review consisted of all population-based studies, ensuring the data were reliable and of high quality and allowing accurate calculation of incidence and survival rate. Second, this review is the latest update on ICH incidence and survival. Third, we performed subgroup analyses on meta-analysis, providing pooled estimates of incidence and survival rate by different country income levels. The present study has several limitations: first, there was significant heterogeneity between the studies reporting incidence of ICH, which cannot be explained by the subgroup analysis on income level or meta-regression on study midyear. The long-range of the study period, the various sample sizes, different regions, age, and ethnicities may contribute to heterogeneity. Two systematic reviews and meta-analyses on stroke and ICH conducted about 10 years ago had significant unexplained heterogeneity across eligible studies (2, 4). Second, our study was based on aggregated data. We have no access to individual data in eligible publications, so we cannot do standardized analysis, age, or sex-based analysis to further investigate the potential sources of heterogeneity. Third, although we have performed a subgroup analysis on income groups, many more studies were from high-income groups than other groups. The imbalance in the distribution of countries by income group may result in less representation of studies from lower-middle income regions. Fourth, ethnicity could be a factor that can explain the difference in the incidence and survival rate. We did not report in different ethnic groups because many countries are multi-ethnic, so that it is ambiguous to classify a country into ethnicity groups. Our future studies could focus on ethnicity differences in ICH incidence and survival. Despite these limitations, this meta-analysis offered a comprehensive, comparable, robust, and latest overview of ICH incidence and long-term survival with population-based studies.

## Conclusion

The pooled ICH incidence was highest in lower-middle-income countries and lowest in HIC. Incidence has not decreased with time. About a half of patients with ICH survived 1 year, and about two-fifths survived 5 years. There were no time trends on survival. Good-quality incidence and survival data can help us improve the prevention of ICH in primary care and its functional outcome and survival in secondary care. More multi-center population-based stroke registers with long follow-up years or surveillance design are needed. They should be continued, especially in low-income and lower-middle-income regions, to provide reliable data on stroke epidemiology. Future studies on ICH should also clarify the case ascertainment based on the brain imaging technique.

## Data Availability Statement

The original contributions presented in the study are included in the article/Supplementary Material, further inquiries can be directed to the corresponding author.

## Author Contributions

XL, CW, and YW: conceptualization. XL and LZ: formal analysis. CW and YW: supervision and writing-review and editing. XL: writing-original draft. All authors contributed to the article and approved the submitted version.

## Funding

This study/project is funded by the National Institute for Health Research (NIHR) [Program Grants for Applied Research (NIHR202339)].

## Author Disclaimer

The views expressed are those of the author(s) and not necessarily those of the NIHR or the Department of Health and Social Care.

## Conflict of Interest

The authors declare that the research was conducted in the absence of any commercial or financial relationships that could be construed as a potential conflict of interest.

## Publisher's Note

All claims expressed in this article are solely those of the authors and do not necessarily represent those of their affiliated organizations, or those of the publisher, the editors and the reviewers. Any product that may be evaluated in this article, or claim that may be made by its manufacturer, is not guaranteed or endorsed by the publisher.
