# Supplementary material for: Incidence and Long-Term Survival of Spontaneous Intracerebral Hemorrhage Over Time: A Systematic Review and Meta-Analysis
Source: Front Neurol. 2022 Mar 10;13:819737. doi: 10.3389/fneur.2022.819737 (PMC8960718; doi:10.3389/fneur.2022.819737)

**Appendix 1 searching strategy**

1. population/ or region.tw. or regional.tw. or population based.tw. or community based.tw. or community.tw. or stroke register.tw. or stroke registry.tw.

2. incidence.tw. or incidence/ or fatality.tw. or mortality.tw. or mortality/ or trend.tw. or survival/

3. hemorrhagic stroke.tw. or stroke/ or intracranial haemorrhage, hypertensive/ or cerebral hemorrhage/ or ((intracerebral or intraparenchymal).tw. and ((hemorrhage or haemorrhage).tw. or hemorrhage/ or hematoma/ or haematoma.tw. or hematoma.tw.))

4. 1 and 2 and 3

5. limit 4 to human

**Appendix 2 Bubble plot of meta regression for study midyear and incidence**


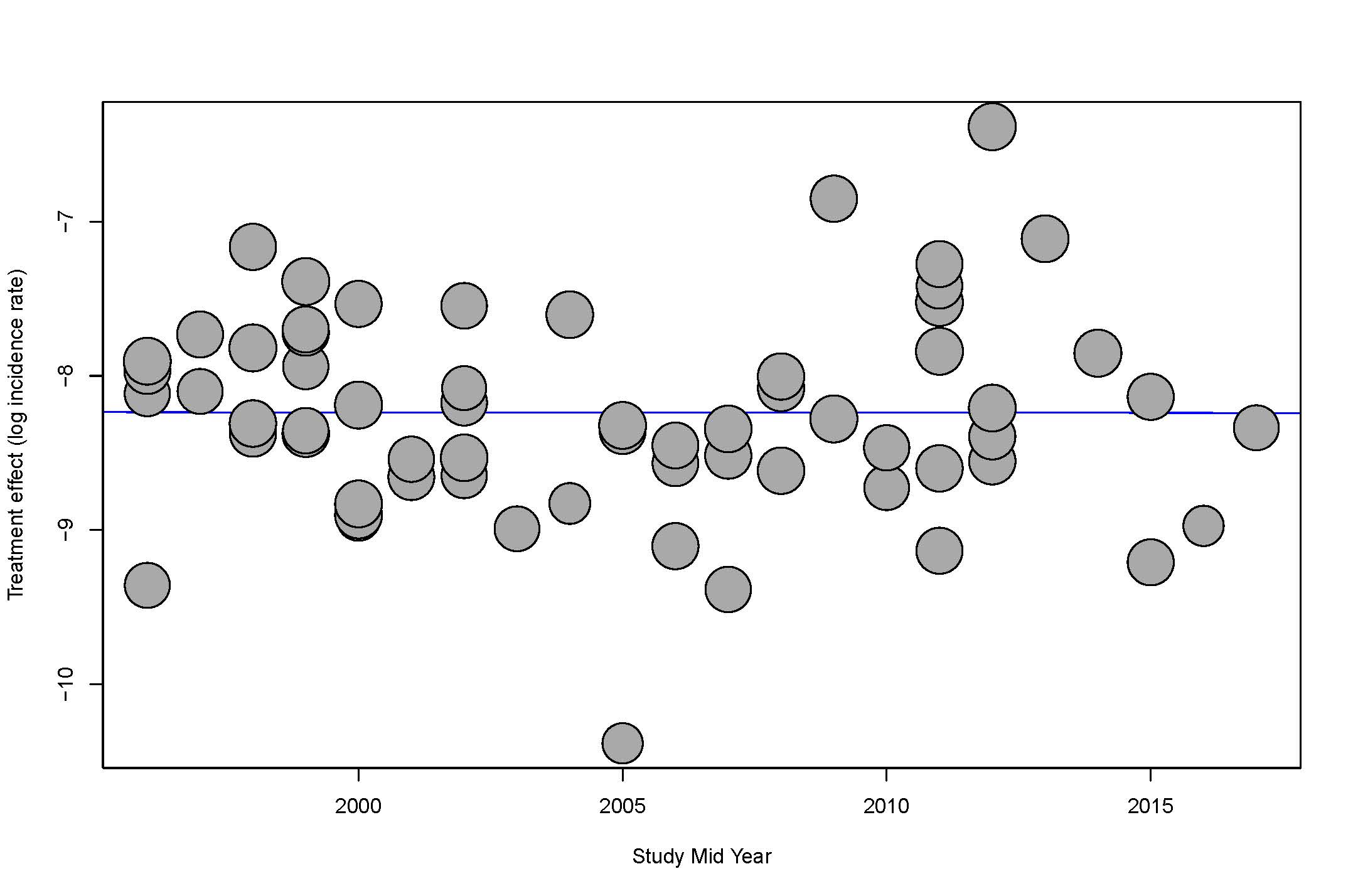


**Appendix 3 Funnel plot of studies reporting incidences after intracerebral haemorrhage**

**
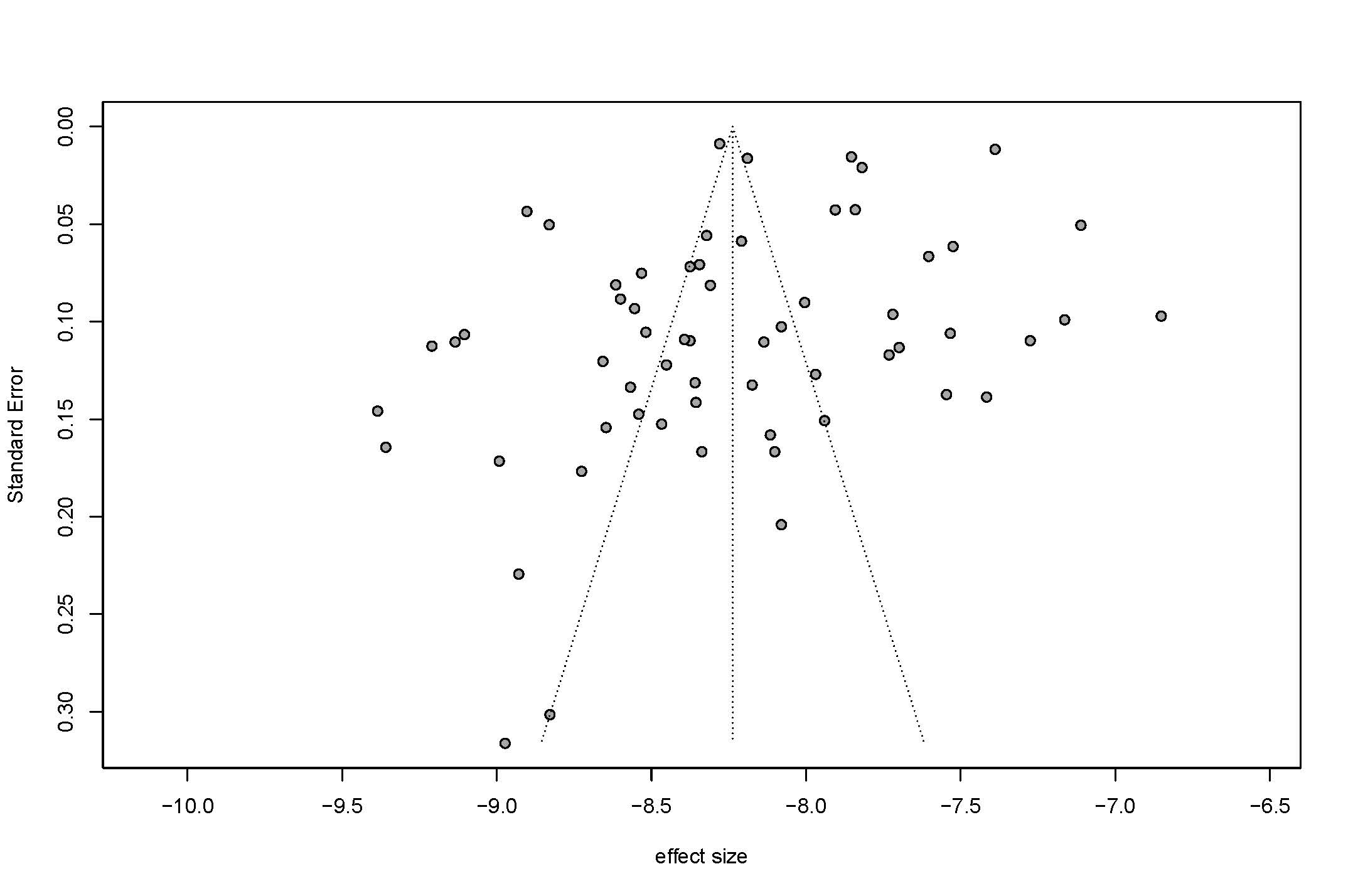
**

**Appendix 4 Funnel plot of studies reporting 1-year survival after intracerebral haemorrhage**


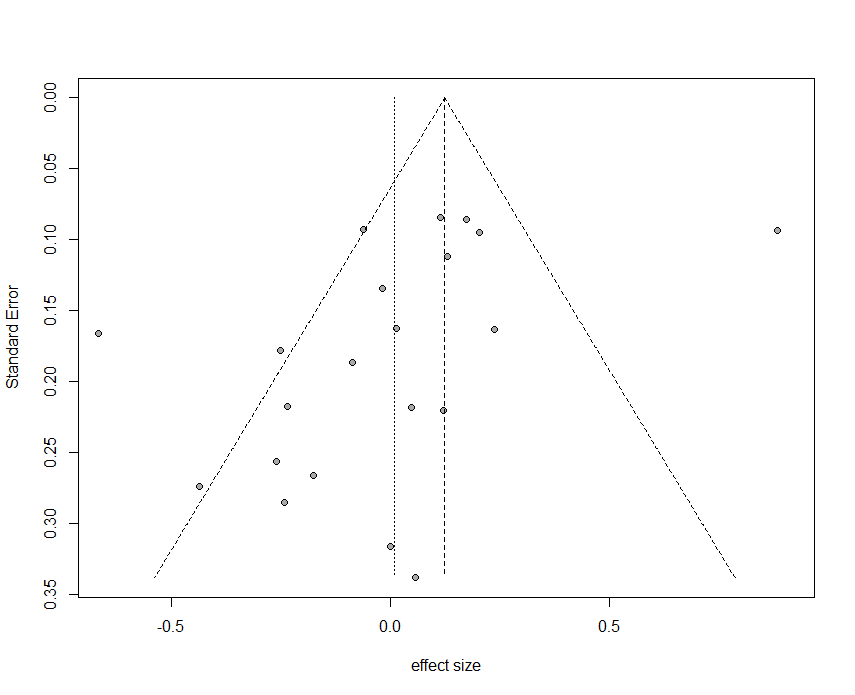


**Appendix 5 Funnel plot of studies reporting 5-year survival after intracerebral haemorrhage**


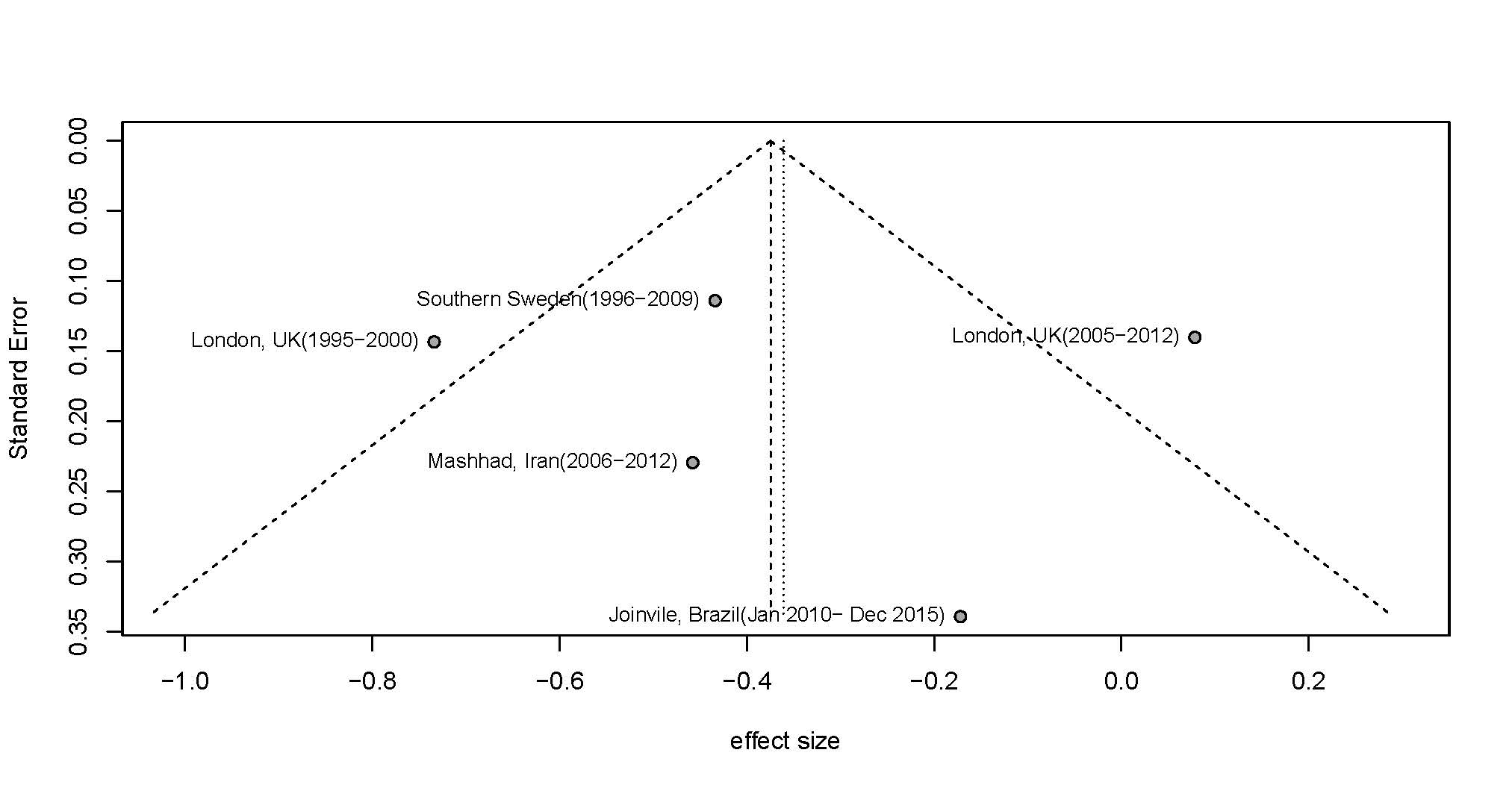

Supplement: Supplementary file 1 [file Table_1.DOCX]
