# Supplementary material for: Incidence and Long-Term Survival of Spontaneous Intracerebral Hemorrhage Over Time: A Systematic Review and Meta-Analysis
Source: Front Neurol. 2022 Mar 10;13:819737. doi: 10.3389/fneur.2022.819737 (PMC8960718; doi:10.3389/fneur.2022.819737)
Supplement: Supplementary file 2 [file Table_2.DOCX]

**Table 1: Quality assessment of the included incidence-related studies according to the Newcastle Ottawa Scale**

| Studies | Selection | | | | Comparability | Outcome | | | Total |
| --- | --- | --- | --- | --- | --- | --- | --- | --- | --- |
|  | Representativeness of the Exposed Cohort (all population-based studies) | Selection of the Non-Exposed Cohort | Ascertainment of Exposure | Demonstration That Outcome of Interest Was Not Present at Start of Study | Comparability of Cohorts on the Basis of the Design or Analysis | Assessment of Outcome (Occurrence of ICH) | Was Follow-Up Long Enough for Outcomes to Occur | Adequacy of Follow Up of Cohorts |  |
| Wolfe, Giroud et al. 2000^1^ | ★ | N/A | N/A | ★ | N/A | ★ | ★ | ★ | 5 |
| Thrift, Dewey et al. 2001^2^ | ★ | N/A | N/A | ★ | N/A | ★ | ★ | / | 4 |
| Di Carlo, Inzitari et al. 2003^3^ | ★ | N/A | N/A | ★ | N/A | ★ | ★ | ★ | 5 |
| Sacco, Marini et al. 2009^4^ Marini, Totaro et al. 2001^5^ | ★ | N/A | N/A | ★ | N/A | ★ | ★ | ★ | 5 |
| D'Alessandro, Bottacchi et al. 2000^6^ | ★ | N/A | N/A | ★ | N/A | ★ | ★ | ★ | 5 |
| Kita, Turin et al. 2009^7^ | ★ | N/A | N/A | ★ | N/A | ★ | ★ | ★ | 5 |
| Ishikawa, Kayaba et al. 2008^8^ | ★ | N/A | N/A | ★ | N/A | ★ | ★ | ★ | 5 |
| Zhang, Yang et al. 2003^9^ | ★ | N/A | N/A | ★ | N/A | ★ | ★ | ★ | 5 |
| Smadja, Cabre et al. 2001^10^ | ★ | N/A | N/A | ★ | N/A | ★ | ★ | ★ | 5 |
| Thrift, Dewey et al. 2009^11^ | ★ | N/A | N/A | ★ | N/A | ★88.7% | ★ | ★ | 5 |
| Correia, Silva et al. 2004^12^ | ★ | N/A | N/A | ★ | N/A | ★ | ★ | ★ | 5 |
| Appelros, Nydevik et al. 2002^13^ | ★ | N/A | N/A | ★ | N/A | ★ | ★ | ★ | 5 |
| Syme, Byrne et al. 2005^14^ | ★ | N/A | N/A | ★ | N/A | ★ | ★ | ★ | 5 |
| Suzuki and Izumi, 2015^15^ | ★ | N/A | N/A | ★ | N/A | ★ | ★ | ★ | 5 |
| Kolominsky-Rabas, Wiedmann et al. 2015^16^ | ★ | N/A | N/A | ★ | N/A | ★ | ★ | ★ | 5 |
| Correia, Magalhaes et al. 2017^17^ | ★ | N/A | N/A | ★ | N/A | / | ★ | ★ | 4 |
| Islam, Anderson et al. 2008^18^ | ★ | N/A | N/A | ★ | N/A | ★ | ★ | ★ | 5 |
| Heuschmann, Grieve et al. 2008^19^  Smeeton, Heuschmann et al. 2007^20^ | ★ | N/A | N/A | ★ | N/A | ★ | ★ | ★ | 5 |
| Giroud, Delpont et al. 2017^21^ | ★ | N/A | N/A | ★ | N/A | ★ | ★ | ★ | 5 |
| Kita, Turin et al. 2009^7^ | ★ | N/A | N/A | ★ | N/A | ★ | ★ | ★ | 5 |
| Vaartjes, Reitsma et al. 2008^22^ | ★ | N/A | N/A | ★ | N/A | / | ★ | ★ | 4 |
| Hallstrom, Jonsson et al. 2007^23^  Hallstrom, Jonsson et al. 2008^24^ | ★ | N/A | N/A | ★ | N/A | ★ | ★ | ★ | 5 |
| Lavados, Sacks et al. 2005^25^ Lavados, Sacks et al. 2010^26^ | ★ | N/A | N/A | ★ | N/A | /(72%) | ★ | ★ | 4 |
| Feigin, Carter et al. 2006^27^ | ★ | N/A | N/A | ★ | N/A | ★ | ★ | ★ | 5 |
| Corbin, Poddar et al. 2004^28^ | ★ | N/A | N/A | ★ | N/A | ★ | ★ | ★ | 5 |
| Manobianca, Zoccolella et al. 2008^29^ | ★ | N/A | N/A | ★ | N/A | ★ | ★ | ★ | 5 |
| Vibo, Korv et al. 2007^30^ | ★ | N/A | N/A | ★ | N/A | ★ | ★ | ★ | 5 |
| Li, Lou et al. 2015^31^ Wang, Bai et al. 2016^32^ Wang, Ning et al. 2014^33^ | ★ | N/A | N/A | ★ | N/A | / | ★ | ★ | 4 |
| Manobianca, Zoccolella et al. 2010^34^ | ★ | N/A | N/A | ★ | N/A | ★ | ★ | ★ | 5 |
| Dalal, Malik et al. 2008^35^ Rothwell, Coull et al. 2004^36^ | ★ | N/A | N/A | ★ | N/A | ★ | ★ | ★ | 5 |
| Minelli, Fen et al. 2007^37^ | ★ | N/A | N/A | ★ | N/A | ★85.2% | ★ | ★ | 5 |
| Carlsson, Wilsgaard et al. 2016^38^ | ★ | N/A | N/A | ★ | N/A | ★ | ★ | ★ | 5 |
| Kleindorfer, Khoury et al. 2010^39^  Kissela, Khoury et al. 2012^40^ | ★ | N/A | N/A | ★ | N/A | ★ | ★ | ★ | 5 |
| Corso, Bottacchi et al. 2009^41^ | ★ | N/A | N/A | ★ | N/A | ★ | ★ | ★ | 5 |
| Groppo, De Gennaro et al. 2012^42^ | ★ | N/A | N/A | ★ | N/A | ★ | ★ | ★ | 5 |
| Dalal, Malik et al. 2008^35^ | ★ | N/A | N/A | ★ | N/A | ★ | ★ | ★ | 5 |
| Cabral, Freire et al. 2017^43^ Cabral, Goncalves et al. 2009^44,45^ | ★ | N/A | N/A | ★ | N/A | ★ | ★ | ★ | 5 |
| Kelly, Crispino et al. 2012^46^ | ★ | N/A | N/A | ★ | N/A | ★ | ★ | ★ | 5 |
| Amiri, Kapral et al. 2018^47^ Azarpazhooh, Etemadi et al. 2010^48^ | ★ | N/A | N/A | ★ | N/A | / | ★ | ★ | 4 |
| Boden-Albala, Allen et al. 2017^49^ | ★ | N/A | N/A | ★ | N/A | ★ | ★ | ★ | 5 |
| Kolominsky-Rabas, Wiedmann et al. 2015^16^ | ★ | N/A | N/A | ★ | N/A | ★ | ★ | ★ | 5 |
| Palm, Henschke et al. 2013^50^ | ★ | N/A | N/A | ★ | N/A | ★ | ★ | ★ | 5 |
| Janes, Gigli et al. 2013^51^ | ★ | N/A | N/A | ★ | N/A | ★ | ★ | ★ | 5 |
| Pikija, Cvetko et al. 2012^52^ | ★ | N/A | N/A | ★ | N/A | ★87% | ★ | ★ | 5 |
| Li, Lou et al. 2015^31^ Wang, Bai et al. 2016^32^ Wang, Ning et al. 2014^33^ | ★ | N/A | N/A | ★ | N/A | / | ★ | ★ | 5 |
| Melina Gattellari et al. 2020^53^ | ★ | N/A | N/A | ★ | N/A | / | ★ | ★ | 4 |
| Neelamegam, Looi et al. 2013^54^ | ★ | N/A | N/A | ★ | N/A | / | ★ | / | 3 |
| Correia, Magalhaes et al. 2017^17^ | ★ | N/A | N/A | ★ | N/A | ★ | ★ | ★ | 5 |
| Cabral, Freire et al. 2017^43^ | ★ | N/A | N/A | ★ | N/A | ★ | ★ | ★ | 5 |
| Stranjalis, Kalamatianos et al. 2014^55^ | ★ | N/A | N/A | ★ | N/A | ★92.4% | ★ | ★ | 5 |
| Tsivgoulis, Patousi et al. 2018^56^ | ★ | N/A | N/A | ★ | N/A | ★ | ★ | ★ | 5 |
| Takashima, Arima et al. 2017^57^ | ★ | N/A | N/A | ★ | N/A | / | ★ | ★ | 4 |
| Samarasekera, Fonville et al. 2015^58^ | ★ | N/A | N/A | ★ | N/A | ★ | ★ | ★ | 5 |
| Okon, Adebobola et al. 2015^59^ | ★ | N/A | N/A | ★ | N/A | ★ | ★ | ★ | 5 |
| Sacco, Ornello et al. 2016^60^ | ★ | N/A | N/A | ★ | N/A | ★ | ★ | ★ | 5 |
| Pandian, Singh et al. 2016^61^ | ★ | N/A | N/A | ★ | N/A | / | ★ | ★ | 4 |
| Olindo, Chausson et al. 2014^62^ | ★ | N/A | N/A | ★ | N/A | ★ | ★ | ★ | 5 |
| Yiping Chen et al. 2020^63^ | ★ | N/A | N/A | ★ | N/A | ★ | ★ | ★ | 5 |
| Wang, Jiang et al. 2017^64^ | ★ | N/A | N/A | ★ | N/A | ★ | ★ | ★ | 5 |
| Walid Saliba et al. 2019^65^ | ★ | N/A | N/A | ★ | N/A | ★ | ★ | ★ | 5 |
| Cabral, Freire et al. 2017^43^ | ★ | N/A | N/A | ★ | N/A | ★ | ★ | ★ | 5 |
| Nzwalo, Nogueira et al. 2017^66^ | ★ | N/A | N/A | ★ | N/A | ★ | ★ | ★ | 5 |
| Cesar Minelli et al. 2020^67^ | ★ | N/A | N/A | ★ | N/A | ★ | ★ | ★ | 5 |
| Peter Appelros et al. 2019^68^ | ★ | N/A | N/A | ★ | N/A | ★ | ★ | ★ | 5 |

**Table 2: Quality assessment of the included survival-related studies according to the Newcastle Ottawa Scale**

| Studies | Selection | | | | Comparability | Outcome | | | Total |
| --- | --- | --- | --- | --- | --- | --- | --- | --- | --- |
|  | Representativeness of the Exposed Cohort (all population-based studies) | Selection of the Non-Exposed Cohort | Ascertainment of Exposure（ICH） | Demonstration That Outcome of Interest Was Not Present at Start of Study | Comparability of Cohorts on the Basis of the Design or Analysis | Assessment of Outcome(Death) | Was Follow-Up Long Enough for Outcomes to Occur | Adequacy of Follow Up of Cohorts |  |
| Thrift et al., 2001^2^ | ★ | N/A | N/A | ★ | N/A | ★ | ★ | ★ | 5 |
| Di Carlo et al., 2003^3^ | ★ | N/A | N/A | ★ | N/A | ★ | ★ | ★ | 5 |
| Sacco et al., 2009^4^ | ★ | N/A | N/A | ★ | N/A | ★ | ★ | ★ | 5 |
| Hillen et al., 2003^69^ | ★ | N/A | N/A | ★ | N/A | ★ | ★ | ★ | 5 |
| Thrift et al., 2009^11^ | ★ | N/A | N/A | ★ | N/A | ★ | ★ | ★ | 5 |
| Syme et al., 2005^14^ | ★ | N/A | N/A | ★ | N/A | ★ | ★ | ★ | 5 |
| Vibo et al., 2007^70^ | ★ | N/A | N/A | ★ | N/A | ★ | ★ | ★ | 5 |
| Hansen et al., 2013^71^ | ★ | N/A | N/A | ★ | N/A | ★ | ★ | ★ | 5 |
| Reem Waziry et al., 2020^72^ | ★ | N/A | N/A | ★ | N/A | ★ | ★ | ★ | 5 |
| McCormick and Chen, 2016^73^ | ★ | N/A | N/A | ★ | N/A | ★ | ★ | ★ | 5 |
| van Beijnum et al., 2009^74^ | ★ | N/A | N/A | ★ | N/A | ★ | ★ | ★ | 5 |
| Shoeibi et al., 2015^75^ | ★ | N/A | N/A | ★ | N/A | ★ | ★ | ★ | 5 |
| Palm et al., 2013^50^ | ★ | N/A | N/A | ★ | N/A | ★ | ★ | ★ | 5 |
| Desikan et al., 2016^76^ | ★ | N/A | N/A | ★ | N/A | ★ | ★ | ★ | 5 |
| Farzadfard et al., 2018^77^ | ★ | N/A | N/A | ★ | N/A | ★ | ★ | ★ | 5 |
| Samarasekera et al., 2015^58^ | ★ | N/A | N/A | ★ | N/A | ★ | ★ | ★ | 5 |
| Oie et al., 2018^78^ | ★ | N/A | N/A | ★ | N/A | ★ | ★ | ★ | 5 |
| Tsivgoulis et al., 2018^79^ | ★ | N/A | N/A | ★ | N/A | ★ | ★ | ★ | 5 |
| Nzwalo et al., 2018^80^ | ★ | N/A | N/A | ★ | N/A | ★ | ★ | ★ | 5 |
| Olindo et al., 2017^81^ | ★ | N/A | N/A | ★ | N/A | ★ | ★ | ★ | 5 |
| Takashima et al., 2018^82^ | ★ | N/A | N/A | ★ | N/A | ★ | ★ | ★ | 5 |
| Sacco et al., 2016^60^ | ★ | N/A | N/A | ★ | N/A | ★ | ★ | ★ | 5 |
| Cabral et al., 2018^83^  Cabral et al., 2015^84^ | ★ | N/A | N/A | ★ | N/A | ★ | ★ | ★ | 5 |

**Reference**

1. Wolfe CD, Giroud M, Kolominsky-Rabas P, et al. Variations in stroke incidence and survival in 3 areas of Europe. European Registries of Stroke (EROS) Collaboration. *Stroke.* 2000;31(9):2074-2079.

2. Thrift AG, Dewey HM, Macdonell RA, McNeil JJ, Donnan GA. Incidence of the major stroke subtypes: initial findings from the North East Melbourne stroke incidence study (NEMESIS). *Stroke.* 2001;32(8):1732-1738.

3. Di Carlo A, Inzitari D, Galati F, et al. A prospective community-based study of stroke in Southern Italy: the Vibo Valentia incidence of stroke study (VISS). Methodology, incidence and case fatality at 28 days, 3 and 12 months. *Cerebrovascular Diseases.* 2003;16(4):410-417.

4. Sacco S, Marini C, Toni D, Olivieri L, Carolei A. Incidence and 10-year survival of intracerebral hemorrhage in a population-based registry. *Stroke.* 2009;40(2):394-399.

5. Marini C, Totaro R, De Santis F, Ciancarelli I, Baldassarre M, Carolei A. Stroke in young adults in the community-based L'Aquila registry: incidence and prognosis. *Stroke.* 2001;32(1):52-56.

6. D'Alessandro G, Bottacchi E, Di Giovanni M, et al. Temporal trends of stroke in Valle d'Aosta, Italy. Incidence and 30-day fatality rates. *Neurological Sciences.* 2000;21(1):13-18.

7. Kita Y, Turin TC, Ichikawa M, et al. Trend of stroke incidence in a Japanese population: Takashima stroke registry, 1990-2001. *International Journal of Stroke.* 2009;4(4):241-249.

8. Ishikawa S, Kayaba K, Gotoh T, et al. Incidence of total stroke, stroke subtypes, and myocardial infarction in the Japanese population: the JMS Cohort Study. *Journal of Epidemiology.* 2008;18(4):144-150.

9. Zhang LF, Yang J, Hong Z, et al. Proportion of different subtypes of stroke in China. *Stroke.* 2003;34(9):2091-2096.

10. Smadja D, Cabre P, May F, et al. ERMANCIA: Epidemiology of Stroke in Martinique, French West Indies: Part I: methodology, incidence, and 30-day case fatality rate. *Stroke.* 2001;32(12):2741-2747.

11. Thrift AG, Dewey HM, Sturm JW, et al. Incidence of stroke subtypes in the North East Melbourne Stroke Incidence Study (NEMESIS): differences between men and women. *Neuroepidemiology.* 2009;32(1):11-18.

12. Correia M, Silva MR, Matos I, et al. Prospective community-based study of stroke in Northern Portugal: incidence and case fatality in rural and urban populations. *Stroke.* 2004;35(9):2048-2053.

13. Appelros P, Nydevik I, Seiger A, Terent A. High incidence rates of stroke in Orebro, Sweden: Further support for regional incidence differences within Scandinavia. *Cerebrovascular Diseases.* 2002;14(3-4):161-168.

14. Syme PD, Byrne AW, Chen R, Devenny R, Forbes JF. Community-based stroke incidence in a Scottish population: the Scottish Borders Stroke Study. *Stroke.* 2005;36(9):1837-1843.

15. Suzuki K, Izumi M. The incidence of hemorrhagic stroke in Japan is twice compared with western countries: the Akita stroke registry. *Neurological Sciences.* 2015;36(1):155-160.

16. Kolominsky-Rabas PL, Wiedmann S, Weingartner M, et al. Time trends in incidence of pathological and etiological stroke subtypes during 16 years: the Erlangen Stroke Project. *Neuroepidemiology.* 2015;44(1):24-29.

17. Correia M, Magalhaes R, Felgueiras R, Quintas C, Guimaraes L, Silva MC. Changes in stroke incidence, outcome, and associated factors in Porto between 1998 and 2011. *International Journal of Stroke.* 2017;12(2):169-179.

18. Islam MS, Anderson CS, Hankey GJ, et al. Trends in incidence and outcome of stroke in Perth, Western Australia during 1989 to 2001: the Perth Community Stroke Study. *Stroke.* 2008;39(3):776-782.

19. Heuschmann PU, Grieve AP, Toschke AM, Rudd AG, Wolfe CD. Ethnic group disparities in 10-year trends in stroke incidence and vascular risk factors: the South London Stroke Register (SLSR). *Stroke.* 2008;39(8):2204-2210.

20. Smeeton NC, Heuschmann PU, Rudd AG, et al. Incidence of hemorrhagic stroke in black Caribbean, black African, and white populations: the South London stroke register, 1995-2004. *Stroke.* 2007;38(12):3133-3138.

21. Giroud M, Delpont B, Daubail B, et al. Temporal Trends in Sex Differences With Regard to Stroke Incidence: The Dijon Stroke Registry (1987-2012). *Stroke.* 2017;48(4):846-849.

22. Vaartjes I, Reitsma JB, de Bruin A, et al. Nationwide incidence of first stroke and TIA in the Netherlands. *European Journal of Neurology.* 2008;15(12):1315-1323.

23. Hallstrom B, Jonsson AC, Nerbrand C, Petersen B, Norrving B, Lindgren A. Lund Stroke Register: hospitalization pattern and yield of different screening methods for first-ever stroke. *Acta Neurologica Scandinavica.* 2007;115(1):49-54.

24. Hallstrom B, Jonsson AC, Nerbrand C, Norrving B, Lindgren A. Stroke incidence and survival in the beginning of the 21st century in southern Sweden: comparisons with the late 20th century and projections into the future. *Stroke.* 2008;39(1):10-15.

25. Lavados PM, Sacks C, Prina L, et al. Incidence, 30-day case-fatality rate, and prognosis of stroke in Iquique, Chile: a 2-year community-based prospective study (PISCIS project). *Lancet.* 2005;365(9478):2206-2215.

26. Lavados PM, Sacks C, Prina L, et al. Incidence of lobar and non-lobar spontaneous intracerebral haemorrhage in a predominantly Hispanic-Mestizo population--the PISCIS stroke project: a community-based prospective study in Iquique, Chile. *Neuroepidemiology.* 2010;34(4):214-221.

27. Feigin V, Carter K, Hackett M, et al. Ethnic disparities in incidence of stroke subtypes: Auckland Regional Community Stroke Study, 2002-2003. *Lancet Neurology.* 2006;5(2):130-139.

28. Corbin DO, Poddar V, Hennis A, et al. Incidence and case fatality rates of first-ever stroke in a black Caribbean population: the Barbados Register of Strokes. *Stroke.* 2004;35(6):1254-1258.

29. Manobianca G, Zoccolella S, Petruzzellis A, Miccoli A, Logroscino G. Low incidence of stroke in southern Italy: a population-based study. *Stroke.* 2008;39(11):2923-2928.

30. Vibo R, Korv J, Roose M. The Third Stroke Registry in Tartu, Estonia, from 2001 to 2003. *Acta Neurologica Scandinavica.* 2007;116(1):31-36.

31. Li B, Lou Y, Gu H, et al. Trends in Incidence of Stroke and Transition of Stroke Subtypes in Rural Tianjin China: A Population-Based Study from 1992 to 2012. *PLoS ONE [Electronic Resource].* 2015;10(10):e0139461.

32. Wang J, Bai L, Shi M, et al. Trends in Age of First-Ever Stroke Following Increased Incidence and Life Expectancy in a Low-Income Chinese Population. *Stroke.* 2016;47(4):929-935.

33. Wang J, Ning X, Yang L, et al. Sex differences in trends of incidence and mortality of first-ever stroke in rural Tianjin, China, from 1992 to 2012. *Stroke.* 2014;45(6):1626-1631.

34. Manobianca G, Zoccolella S, Petruzzellis A, Miccoli A, Logroscino G. The incidence of major stroke subtypes in southern Italy: a population-based study. *European Journal of Neurology.* 2010;17(9):1148-1155.

35. Dalal PM, Malik S, Bhattacharjee M, et al. Population-based stroke survey in Mumbai, India: incidence and 28-day case fatality. *Neuroepidemiology.* 2008;31(4):254-261.

36. Rothwell PM, Coull AJ, Giles MF, et al. Change in stroke incidence, mortality, case-fatality, severity, and risk factors in Oxfordshire, UK from 1981 to 2004 (Oxford Vascular Study). *Lancet.* 2004;363(9425):1925-1933.

37. Minelli C, Fen LF, Minelli DP. Stroke incidence, prognosis, 30-day, and 1-year case fatality rates in Matao, Brazil: a population-based prospective study. *Stroke.* 2007;38(11):2906-2911.

38. Carlsson M, Wilsgaard T, Johnsen SH, et al. Temporal Trends in Incidence and Case Fatality of Intracerebral Hemorrhage: The Tromso Study 1995-2012. *Cerebrovascular Diseases Extra.* 2016;6(2):40-49.

39. Kleindorfer DO, Khoury J, Moomaw CJ, et al. Stroke incidence is decreasing in whites but not in blacks: a population-based estimate of temporal trends in stroke incidence from the Greater Cincinnati/Northern Kentucky Stroke Study. *Stroke.* 2010;41(7):1326-1331.

40. Kissela BM, Khoury JC, Alwell K, et al. Age at stroke: temporal trends in stroke incidence in a large, biracial population. *Neurology.* 2012;79(17):1781-1787.

41. Corso G, Bottacchi E, Giardini G, et al. Community-based study of stroke incidence in the Valley of Aosta, Italy. CARe-cerebrovascular Aosta Registry: years 2004-2005. *Neuroepidemiology.* 2009;32(3):186-195.

42. Groppo E, De Gennaro R, Granieri G, et al. Incidence and prognosis of stroke in young adults: a population-based study in Ferrara, Italy. *Neurological Sciences.* 2012;33(1):53-58.

43. Cabral NL, Freire AT, Conforto AB, et al. Increase of Stroke Incidence in Young Adults in a Middle-Income Country: A 10-Year Population-Based Study. *Stroke.* 2017;48(11):2925-2930.

44. Cabral NL, Goncalves AR, Longo AL, et al. Incidence of stroke subtypes, prognosis and prevalence of risk factors in Joinville, Brazil: a 2 year community based study. *Journal of Neurology, Neurosurgery & Psychiatry.* 2009;80(7):755-761.

45. Cabral NL, Goncalves AR, Longo AL, et al. Trends in stroke incidence, mortality and case fatality rates in Joinville, Brazil: 1995-2006. *Journal of Neurology, Neurosurgery & Psychiatry.* 2009;80(7):749-754.

46. Kelly PJ, Crispino G, Sheehan O, et al. Incidence, event rates, and early outcome of stroke in Dublin, Ireland: the North Dublin population stroke study. *Stroke.* 2012;43(8):2042-2047.

47. Amiri A, Kapral MK, Thrift AG, et al. The Incidence and Characteristics of Stroke in Urban-Dwelling Iranian Women. *Journal of Stroke & Cerebrovascular Diseases.* 2018;27(3):547-554.

48. Azarpazhooh MR, Etemadi MM, Donnan GA, et al. Excessive incidence of stroke in Iran: evidence from the Mashhad Stroke Incidence Study (MSIS), a population-based study of stroke in the Middle East. *Stroke.* 2010;41(1):e3-e10.

49. Boden-Albala B, Allen J, Roberts ET, Bulkow L, Trimble B. Ascertainment of Alaska Native Stroke Incidence, 2005-2009: Lessons for Assessing the Global Burden of Stroke. *Journal of Stroke & Cerebrovascular Diseases.* 2017;26(9):2019-2026.

50. Palm F, Henschke N, Wolf J, et al. Intracerebral haemorrhage in a population-based stroke registry (LuSSt): incidence, aetiology, functional outcome and mortality. *Journal of Neurology.* 2013;260(10):2541-2550.

51. Janes F, Gigli GL, D'Anna L, et al. Stroke incidence and 30-day and six-month case fatality rates in Udine, Italy: a population-based prospective study. *International Journal of Stroke.* 2013;8 Suppl A100:100-105.

52. Pikija S, Cvetko D, Malojcic B, et al. A population-based prospective 24-month study of stroke: incidence and 30-day case-fatality rates of first-ever strokes in Croatia. *Neuroepidemiology.* 2012;38(3):164-171.

53. Gattellari M, Goumas C, Jalaludin B, Worthington JM. Population-based stroke surveillance using big data: state-wide epidemiological trends in admissions and mortality in New South Wales, Australia. *Neurol Res.* 2020;42(7):587-596.

54. Neelamegam M, Looi I, Cheah WK, Narayanan P, Hamid AM, Ong LM. Stroke incidence in the South West District of the Penang Island, Malaysia: PEARLs: Penang Acute Stroke Research Longitudinal Study. *Preventive Medicine.* 2013;57 Suppl:S77-79.

55. Stranjalis G, Kalamatianos T, Gatzonis S, Loufardaki M, Tzavara C, Sakas DE. The incidence of the first-ever stroke in a Mediterranean island population: the isle of Lesvos stroke study. *Neuroepidemiology.* 2014;43(3-4):206-212.

56. Tsivgoulis G, Patousi A, Pikilidou M, et al. Stroke Incidence and Outcomes in Northeastern Greece: The Evros Stroke Registry. *Stroke.* 2018;49(2):288-295.

57. Takashima N, Arima H, Kita Y, et al. Incidence, Management and Short-Term Outcome of Stroke in a General Population of 1.4 Million Japanese - Shiga Stroke Registry. *Circulation Journal.* 2017;81(11):1636-1646.

58. Samarasekera N, Fonville A, Lerpiniere C, et al. Influence of intracerebral hemorrhage location on incidence, characteristics, and outcome: population-based study. *Stroke.* 2015;46(2):361-368.

59. Okon M, Adebobola NI, Julius S, et al. Stroke incidence and case fatality rate in an urban population. *Journal of Stroke & Cerebrovascular Diseases.* 2015;24(4):771-777.

60. Sacco S, Ornello R, Degan D, Tiseo C, Pistoia F, Carolei A. Declining incidence of intracerebral hemorrhage over two decades in a population-based study. *European Journal of Neurology.* 2016;23(11):1627-1634.

61. Pandian JD, Singh G, Kaur P, et al. Incidence, short-term outcome, and spatial distribution of stroke patients in Ludhiana, India. *Neurology.* 2016;86(5):425-433.

62. Olindo S, Chausson N, Mejdoubi M, et al. Trends in incidence and early outcomes in a Black Afro-Caribbean population from 1999 to 2012: Etude Realisee en Martinique et Centree sur l'Incidence des Accidents Vasculaires Cerebraux II Study. *Stroke.* 2014;45(11):3367-3373.

63. Chen Y, Wright N, Guo Y, et al. Mortality and recurrent vascular events after first incident stroke: a 9-year community-based study of 0.5 million Chinese adults. *Lancet Glob Health.* 2020;8(4):e580-e590.

64. Wang W, Jiang B, Sun H, et al. Prevalence, Incidence, and Mortality of Stroke in China: Results from a Nationwide Population-Based Survey of 480 687 Adults. *Circulation.* 2017;135(8):759-771.

65. Saliba W, Barnett-Griness O, Gronich N, et al. Association of Diabetes and Glycated Hemoglobin With the Risk of Intracerebral Hemorrhage: A Population-Based Cohort Study. *Diabetes Care.* 2019;42(4):682-688.

66. Nzwalo H, Nogueira J, Felix C, et al. Incidence and case-fatality from spontaneous intracerebral hemorrhage in a southern region of Portugal. *Journal of the Neurological Sciences.* 2017;380:74-78.

67. Minelli C, Cabral NL, Ujikawa LT, et al. Trends in the Incidence and Mortality of Stroke in Matao, Brazil: The Matao Preventing Stroke (MAPS) Study. *Neuroepidemiology.* 2020;54(1):75-82.

68. Appelros P. Secular Trends of Stroke Epidemiology in Orebro, Sweden, 2017 Compared to the Trends in 1999: A Population-Based Study. *Cerebrovasc Dis.* 2019;48(3-6):149-156.

69. Hillen T, Coshall C, Tilling K, et al. Cause of stroke recurrence is multifactorial: patterns, risk factors, and outcomes of stroke recurrence in the South London Stroke Register. *Stroke.* 2003;34(6):1457-1463.

70. Vibo R, Korv J, Roose M. One-year outcome after first-ever stroke according to stroke subtype, severity, risk factors and pre-stroke treatment. A population-based study from Tartu, Estonia. *European Journal of Neurology.* 2007;14(4):435-439.

71. Hansen BM, Nilsson OG, Anderson H, Norrving B, Saveland H, Lindgren A. Long term (13 years) prognosis after primary intracerebral haemorrhage: a prospective population based study of long term mortality, prognostic factors and causes of death. *Journal of Neurology, Neurosurgery & Psychiatry.* 2013;84(10):1150-1155.

72. Waziry R, Heshmatollah A, Bos D, et al. Time Trends in Survival Following First Hemorrhagic or Ischemic Stroke Between 1991 and 2015 in the Rotterdam Study. *Stroke.* 2020;51(3):STROKEAHA119027198.

73. McCormick J, Chen R. Impact of socioeconomic deprivation on mortality in people with haemorrhagic stroke: a population-based cohort study. *Postgraduate medical journal.* 2016;92(1091):501-505.

74. van Beijnum J, Lovelock CE, Cordonnier C, et al. Outcome after spontaneous and arteriovenous malformation-related intracerebral haemorrhage: population-based studies. *Brain.* 2009;132(Pt 2):537-543.

75. Shoeibi A, Salehi M, Thrift AG, et al. One‐year case fatality rate following stroke in the M ashhad S troke I ncidence S tudy: a population‐based study of stroke in I ran. *International Journal of Stroke.* 2015;10:96-102.

76. Desikan A, Crichton S, Hoang U, et al. Effect of Exhaust- and Nonexhaust-Related Components of Particulate Matter on Long-Term Survival After Stroke. *Stroke.* 2016;47(12):2916-2922.

77. Farzadfard MT, Thrift AG, Amiri A, et al. Five-Year Case Fatality Following First-Ever Stroke in the Mashhad Stroke Incidence Study: A Population-Based Study of Stroke in the Middle East. *Journal of Stroke & Cerebrovascular Diseases.* 2018;27(4):1085-1089.

78. Oie LR, Madsbu MA, Solheim O, et al. Functional outcome and survival following spontaneous intracerebral hemorrhage: A retrospective population-based study. *Brain and Behavior.* 2018;8(10):e01113.

79. Tsivgoulis G, Katsanos AH, Patousi A, et al. Stroke recurrence and mortality in northeastern Greece: the Evros Stroke Registry. *Journal of neurology.* 2018;265(10):2379-2387.

80. Nzwalo H, Felix C, Nogueira J, et al. Predictors of long-term survival after spontaneous intracerebral hemorrhage in southern Portugal: A retrospective study of a community representative population. *Journal of the Neurological Sciences.* 2018;394:122-126.

81. Olindo S, Saint-Vil M, Jeannin S, et al. One-year disability, death and recurrence after first-ever stroke in a Black Afro-Caribbean population. *International Journal of Stroke.* 2017;12(8):844-850.

82. Takashima N, Arima H, Kita Y, et al. Two-Year Survival After First-Ever Stroke in a General Population of 1.4 Million Japanese―Shiga Stroke Registry―. *Circulation Journal.* 2018;82(10):2549-2556.

83. Cabral NL, Nagel V, Conforto AB, et al. Five-year survival, disability, and recurrence after first-ever stroke in a middle-income country: A population-based study in Joinvile, Brazil. *International Journal of Stroke.* 2018;13(7):725-733.

84. Cabral NL, Muller M, Franco SC, et al. Three-year survival and recurrence after first-ever stroke: the Joinville stroke registry. *BMC Neurology.* 2015;15:70.
